# Supplementary material for: Metformin and Everolimus: A Promising Combination for Neuroendocrine Tumors Treatment
Source: Cancers (Basel). 2020 Aug 2;12(8):2143. doi: 10.3390/cancers12082143 (PMC7464161; doi:10.3390/cancers12082143)
Supplement: Supplementary file 1 [file cancers-12-02143-s001.pdf]

cancers

Supplementary Materials: Metformin and Everolimus: A Promising Combination for Neuroendocrine Tumors Treatment

Eleonora Vitali, Ilena Boemi, Giulia Tarantola, Sara Piccini, Alessandro Zerbi, Giulia Veronesi, Roberto Baldelli, Gherardo Mazziotti, Valeria Smiroldo, Elisabetta Lavezzi, Anna Spada, Giovanna Mantovani and Andrea G. Lania

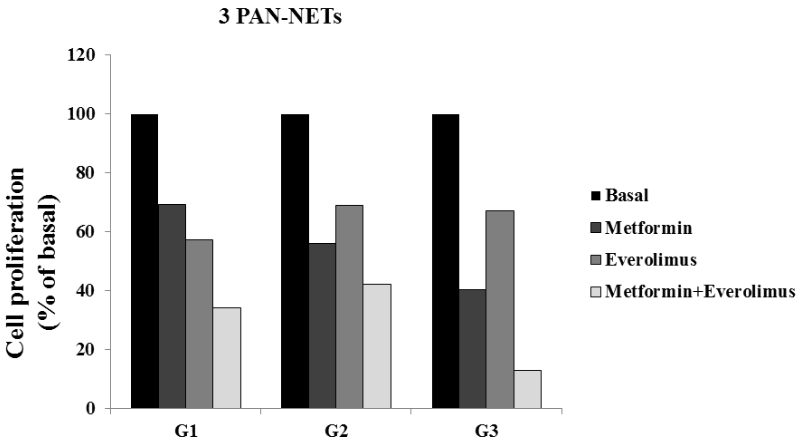

Figure S1. Cell proliferation of three PA-NETs. The graph represents the effect of metformin (10 mM) and everolimus (10 nM) on cell proliferation of three different PAN-NETs (G1-G2-G3).

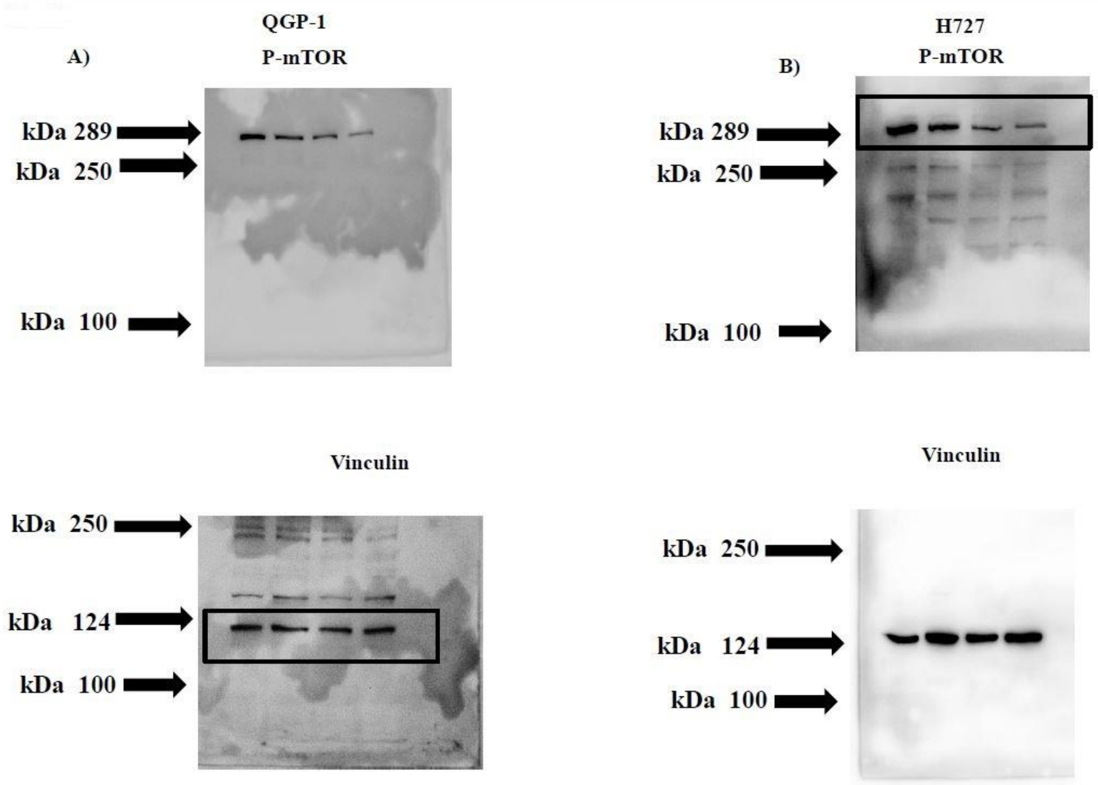

Figure S2. Unprocessed images for WB results (P mTOR and Vinculin) of QGP 1 and H727 cells, corresponding to the Figures 5A and 5B.

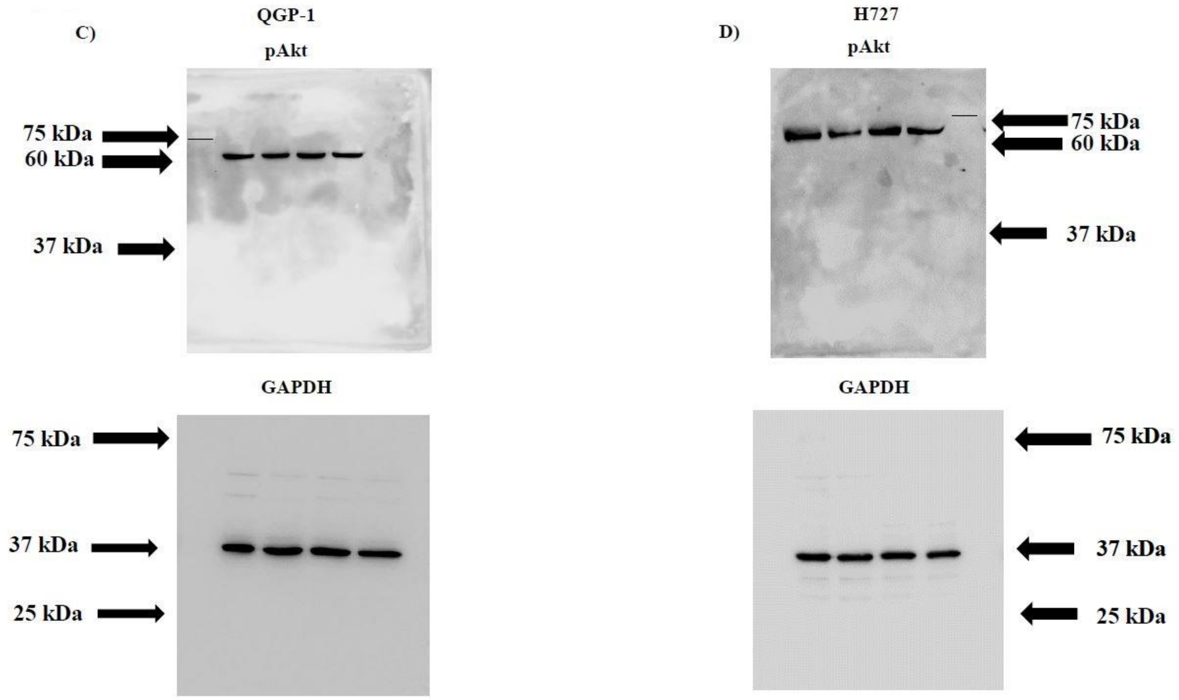

Figure S3. Unprocessed images for WB results (pAktand GAPDH) of QGP-1 and H727 cells, corresponding to the Figures 5C and 5D.

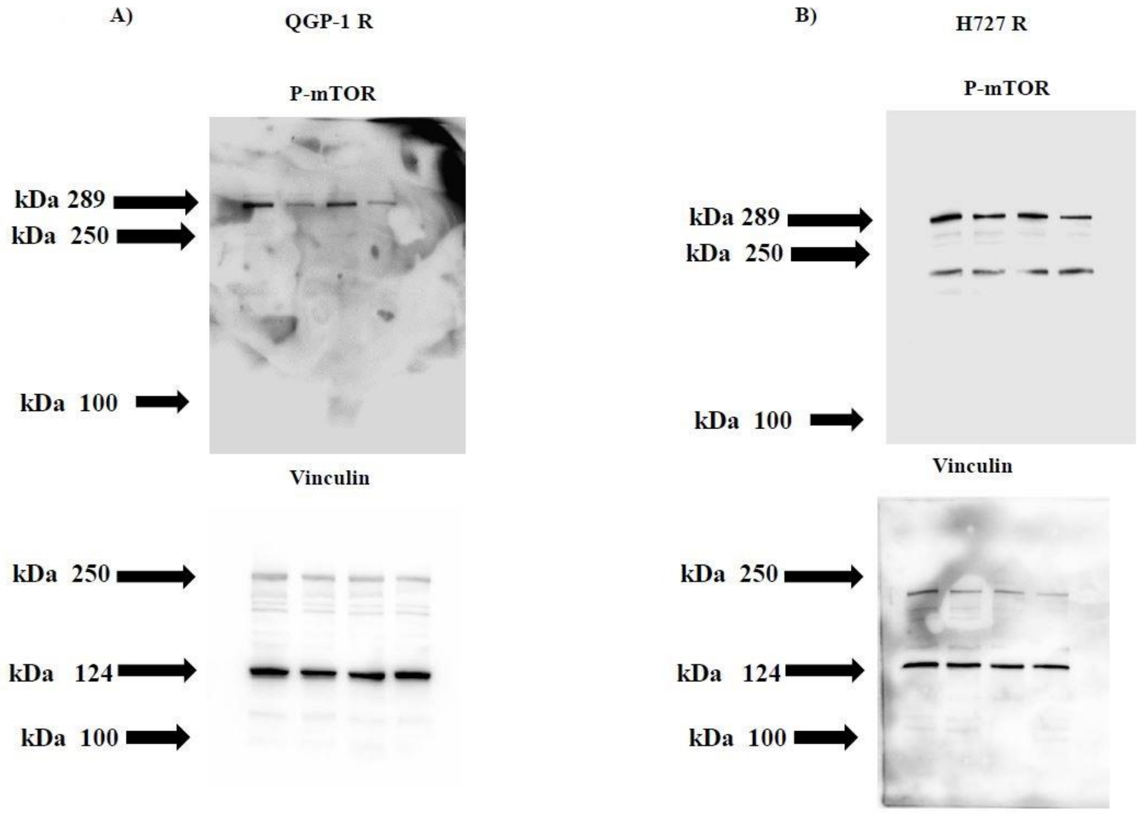

Figure S4. Unprocessed images for WB results (P-mTORand Vinculin) of QGP-1 R and H727 R cells, corresponding to the Figures 7A and 7B.

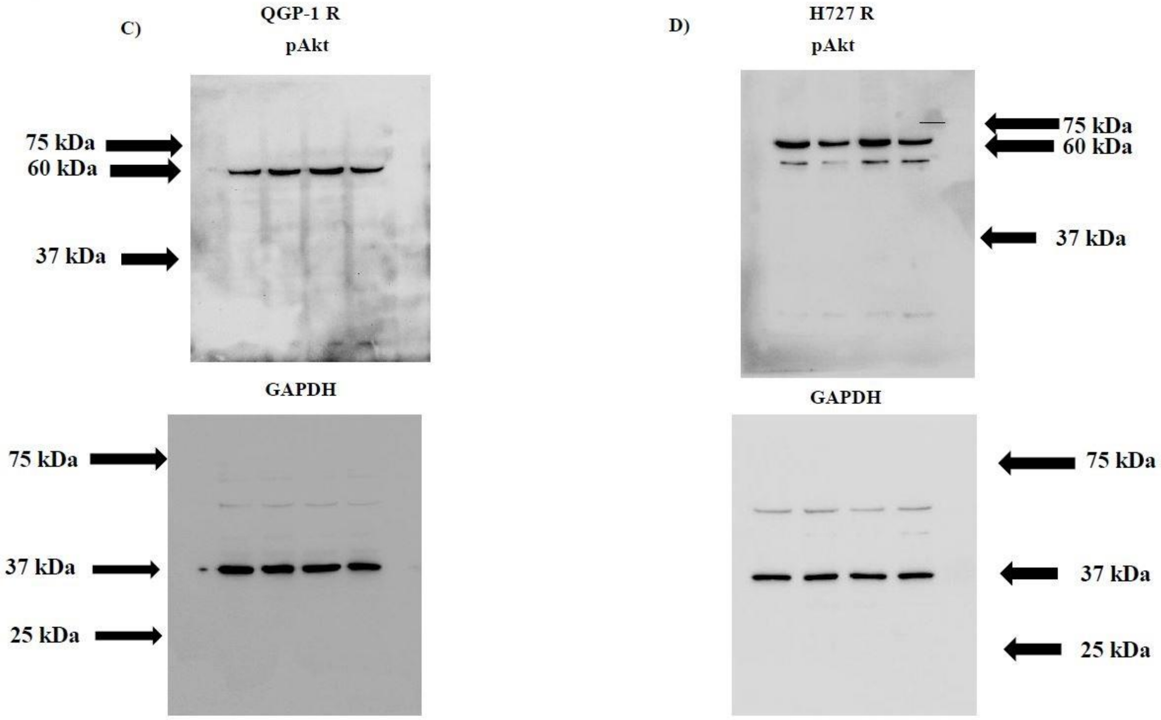

Figure S5. Unprocessed images for WB results (pAktand GAPDH) of QGP-1 R and H727 cells, corresponding to the Figures 7C and 7D.

Table S1. Pathological Findings of PAN-NETs and PNTs included in the study.

Case Number	Diagnosis	Sex	Age	Grade	Ki-67 %	IHC	Location	Stage at Diagnosis	Medical Treatments Before Surgery
1	PAN-NET	F	60	G2	7	CgA (+), synaptophisin (+), CD56 (+, weak)	PANCREATIC BODY	pT2 N0 R0	None
2	PAN-NET	F	34	G3	30	CgA (+), synaptophisin (+), CD56 (+)	PANCREATIC NECK	pT2 N1 R0	None
3	PAN-NET	F	39	G1	1	CgA (+), synaptophisin (+), CD56 (+)	PANCREATIC BODY	pT3 N0 M0	None
4	Typical PNT	F	76	Low	MIB1 5%	CgA (+), synaptophisin (+)	RIGHT LUNG, MIDDLE LOBE	pT2a; N0	None
5	Typical PNT	M	72	Low	MIB1 5%	TTF1(−), CgA (+), synaptophisin (+)	RIGHT LUNG, MIDDLE LOBE	pT2b; N0	None
6	Typical PNT	M	54	Low	MIB1 1%	TTF1(−), CgA (+), synaptophisin (+)	RIGHT LUNG, SUPERIOR LOBE	pT1b N0 R0	None

© 2020 by the authors. Licensee MDPI, Basel, Switzerland. This article is an open access article distributed under the terms and conditions of the Creative Commons Attribution (CC BY) license (http://creativecommons.org/licenses/by/4.0/).
